# Soluble CTLA-4 and high-risk genetic variants: A new frontier in pancreatic ductal adenocarcinoma (PDAC) biomarkers

**DOI:** 10.1016/j.bbrep.2025.102304

**Published:** 2025-10-14

**Authors:** Marwa Hassan, Yasmine Elhusseny, Fatma Elbatol Agamy, Mariam Magdy Azmy, Mahmoud Balata

**Affiliations:** a Immunology Department, Theodor Bilharz Research Institute, Giza, Egypt; b Medical Biochemistry and Molecular Biology, School of Medicine, NewGiza University, Giza, Egypt; c Faculty of Medicine, Al-Azhar university, Cairo, Egypt; d Faculty of Biotechnology, Misr University for Science and Technology, Giza, Egypt; e University Hospital Rostock, Ernst-Heydemann-Straße 6, 18057, Rostock, Germany; f University Hospital Bonn, Venusberg-Campus 1, 53127, Bonn, Germany

**Keywords:** CTLA-4, Gene polymorphisms, Immune checkpoint, Pancreatic ductal adenocarcinoma, rs11571317, rs231726

## Abstract

**Background:**

CTLA-4 is regarded as the “leader” of immune checkpoint inhibitors, as it suppresses autoreactive T cells at the initial phase of naïve T-cell activation. Its genetic variability may impair its effectiveness in the anti-tumor response. Additionally, the presence of soluble CTLA-4 (sCTLA-4) in the tumor microenvironment suppresses effector T-cell activation, allowing for tumor evasion. Therefore, this study aimed to explore the relationship between CTLA-4 polymorphisms and sCTLA-4 and the risk of pancreatic ductal adenocarcinoma (PDAC) in an Egyptian population.

**Method:**

Three single-nucleotide polymorphisms of the CTLA-4 gene (rs231726, rs11571317, and rs13384548) were analyzed, and sCTLA-4 levels were assessed in 125 PDAC patients and 125 healthy controls.

**Results:**

The rs231726 TT genotype was associated with a six-fold greater risk of PDAC, advanced disease grades, and elevated carbohydrate antigen 19-9 (CA19-9) and carcinoembryonic antigen (CEA). Also, the T-allele increased the likelihood of developing PDAC by four times. Conversely, the CC genotype and C-allele conferred protection against PDAC development. The rs11571317 CC genotype and C-allele elevated the PDAC risk by 4.91 and 3.49-fold, respectively, with no relationship to disease severity. The rs13384548 polymorphism showed no significant association with PDAC susceptibility. sCTLA-4 correlated positively with CA19-9 and CEA levels, with the highest levels found in individuals possessing the rs231726 TT genotype and high malignancy grades.

**Conclusions:**

These findings highlight the potential of CTLA-4 SNPs and sCTLA-4 as biomarkers for PDAC diagnosis and prognosis. Integrating these biomarkers into screening programs can offer improved strategies for early detection and disease management in high-risk populations.

## Highlights

• Rs231726 TT genotype and T-allele are linked to a high risk of developing PDAC.
• Rs231726 TT genotype is associated with advanced clinical grading.
• Rs11571317 CC genotype and C-allele cause an increased risk of PDAC.
• SCTLA-4 is increased in PDAC patients with the highest levels in grade 3 illness.
• SCTLA-4 correlates positively with CA19-9 and CEA levels

## 1 Introduction

Pancreatic cancer (PC) has recently been designated a medical emergency by the United European Gastroenterology, ranking as the seventh leading cause of cancer-related mortality worldwide [1]. The disease is characterized by high invasiveness and dismal prognosis, largely due to its silent nature, causing patients to be diagnosed at an advanced stage. Pancreatic ductal adenocarcinoma (PDAC) is the predominant form of PC, with a five-year survival rate of approximately 10 % owing to delayed diagnosis and the lack of efficient therapeutic options [2,3]. Conversely, the five-year survival rate for individuals with surgically resectable PDAC is ∼44 % [4]. Consequently, there has been an explosion in research investigating candidate biomarkers over the past decade, with the expectation that a diagnostic biomarker may offer a solution to this clinical challenge [5,6].

Cytotoxic T lymphocyte–associated antigen 4 (CTLA-4 or CD152), a transmembrane glycoprotein, is a crucial inhibitory molecule expressed by activated T lymphocytes. It plays an essential role in the modulation of the cell cycle, regulation of T-cell proliferation, and production of cytokines [7,8]. It is homologous to CD28, a costimulatory molecule found on the surface of naïve T cells that enables the activation and proliferation of T cells. Both CD28 and CTLA-4 bind to B7 family molecules, including CD80 and CD86, which are expressed on the surface of antigen-presenting cells (APCs). However, CTLA-4 possesses a 10- to 100-fold superior affinity than CD28 for binding CD80 and CD86 [9]. Upon T-cell activation, CTLA-4 is translocated to the cell membrane by exocytosis of CTLA-4-containing vesicles, overtaking B7 from CD28, thereby blocking the second signal required for T-cell activation [10]. Also, CTLA-4 may eliminate CD80 and CD86 from the surfaces of APCs through *trans*-endocytosis, limiting the accessibility of these stimulatory molecules to other CD28-expressing cells [11,12]. This leads to T cells anergy and clonal tolerance, which also inhibits dendritic cells (DCs) differentiation [13].

In the context of chronic inflammation, T cells exhibit exhaustion and upregulate many inhibitory receptors, including PD-1, CTLA-4, or lymphocyte-activation gene 3 (LAG-3), which diminish their efficacy. Initially recognized in chronic viral infection, where the host can't mount an effective immune response and clear the pathogen, it is now evident that exhausted T cells may also arise in malignancies [14]. Under these conditions, it is thought that sustained high antigenic load stimulates T cells to express the inhibitory receptors, whose signaling causes a subsequent progressive decline in the proliferative capacity and effector functions and, in certain instances, their deletion [15].

The CTLA-4 gene is located on chromosome 2q33, and many single-nucleotide polymorphisms (SNPs) of this gene have been identified [7]. These polymorphisms may influence gene expression and are associated with increased susceptibility to various malignancies, including breast cancer, hepatocellular carcinoma, cervical cancer, colorectal cancer, and melanoma, among others [16]. Nevertheless, some research studies have shown an absence of association between CTLA-4 polymorphisms and certain cancer types in particular populations [17,18]. Understanding the CTLA-4 polymorphisms may offer valuable insights for patient screening and the tailored implementation of immunotherapy in oncology [19].

While CTLA-4 SNPs have been examined in several malignancies, their contribution to PDAC has not been previously addressed [20]. To date, no study has concurrently assessed both CTLA-4 genetic variants and circulating soluble CTLA-4 (sCTLA-4) levels in PDAC patients. Considering the aggressive nature of PDAC, the lack of reliable genetic biomarkers, and the emerging role of immune checkpoint pathways in guiding immunotherapy, exploring CTLA-4 in this context is a novel and clinically relevant approach. Accordingly, this study was conducted to investigate the association between the CTLA-4 genetic variants (rs231726, rs11571317, and rs13384548) and the serological levels of sCTLA-4 and the risk of developing PDAC in the Egyptian population.

## 2 Materials and methods

### 2.1 Subjects of the study

A total of 125 patients having PDAC were recruited from the Hepato-Gastroenterology Department at Theodor Bilharz Research Institute, along with 125 age- and sex-matched healthy volunteers as controls for this study. The study received approval from the Ethics Committee of Theodor Bilharz Research Institute (TBRI) (FWA 0010609; PT 797) in accordance with the guidelines of the 1975 Declaration of Helsinki. Informed written consents were obtained from all participants, as indicated by the TBRI Ethics Committee. All patients met the following criteria: (a) aged above 18; (b) diagnosed with PDAC based on a combination of clinical, biochemical, and imaging data.

All the participants underwent a comprehensive medical history assessment focusing on smoking history, previous intervention, and drug intake, thorough general and abdominal examinations, laboratory investigations including liver and kidney function tests and measurements of carbohydrate antigen 19-9 (CA19-9) and carcinoembryonic antigen (CEA), and abdominal ultrasound examination. A CT (TOSHIBA, Japan) scan of the abdomen and pelvis was performed to confirm the presence of a pancreatic lesion. For the staging of pancreatic cancer, an endoscopic ultrasound (EUS) was conducted with the Linear EUS EG-3870UTK Ultrasound Video Endoscopy system from PENTAX (3.8), Japan. Tissue diagnosis was performed using the EUS-fine needle aspiration (FNA) (EUS FNA19 and 22 Gauge, USA) and biopsy core in cases of suspected malignancies of the pancreatic head and distal biliary duct lesions to confirm the final diagnosis.

The staging of cancer was determined by the presence of an ill-defined pancreatic lesion with heterogeneous enhancement, possible invasion of the adjacent structures, such as blood vessels, common bile duct, and duodenum, as well as lymphadenopathy, which appears as large, rounded, heterogeneous, hypoechoic masses with poor differentiation between cortex and medulla and central necrosis.

Patients exhibiting clinical or laboratory indications of other malignancies or those who declined participation in the study were excluded from the study.

### 2.2 Measurement of sCTLA-4 level

Five milliliters of blood were collected from each subject. Two milliliters were subjected to centrifugation to separate serum. The sCTLA-4 level in serum was quantified utilizing the Human CTLA-4 (Soluble) ELISA Kit (Thermo Fisher Scientific, USA). The ELISA analysis was carried out according to the manufacturer's protocol.

### 2.3 DNA extraction and SNP genotyping

Genomic DNA was extracted from the collected blood samples with the GeneJET Genomic DNA Purification Kit (Thermo Fisher Scientific, USA) following the manufacturer's guidelines. The isolated DNA was stored at −80 °C for subsequent utilization. Three SNPs of the CTLA-4 gene, namely rs231726 (C > T), rs11571317 (C > T), and rs13384548 (G > A), were analyzed by real-time PCR. The selection of SNPs was dependent on their confirmed association with disease or their demonstrated functional importance. The PCR was performed using TaqMan™ Genotyping Master Mix (Thermo Fisher Scientific, USA) and TaqMan™ SNP Genotyping Assay (40X) (Thermo Fisher Scientific, USA), complying with the manufacturer's instructions. A negative control was prepared for each SNP as well. The real-time PCR cycling was conducted on the StepOnePlus Real-Time PCR System (Thermo Fisher Scientific, USA). VIC and FAM served as probes for the alleles [VIC/FAM]: rs231726 [C/T], rs11571317 [C/T], and rs13384548 [G/A].

### 2.4 Statistical analysis

The results were analyzed using the SPSS statistical software package version 25 (SPSS Inc., Chicago, IL). All the quantitative variables were presented as mean and standard error (SE), whilst the qualitative variables were reported as frequency and percentage. The Shapiro-Wilk test was used to ascertain the normal distribution of the data. According to the results of normality testing, an Independent Samples *t*-test was employed for normally distributed data, whereas the Mann-Whitney *U* test was applied for non-normally distributed variables. The Chi-square test was used to compare proportions among the categorical variables across the groups. An ANOVA test, followed by LSD, was conducted to compare each set of three groups of quantitative variables. The Hardy-Weinberg test was used to assess the heredity equilibrium. The odds ratio (OR) with a 95 % confidence interval determined the genotype relative risk. A post hoc power analysis was conducted to determine the achieved power for the primary genetic associations found in this study. It indicated that the study had more than 85 % power to detect associations at a significance level of 0.05 for the examined SNPs. The Spearman correlations were done to identify the relationships among the quantitative variables. All statistical analyses yielded significant results at a 0.05 significance level (*p*<0.05).

## 3 Results

### 3.1 Characteristics of the study participants

There were 250 participants in this study, comprising 157 males and 93 females, ranging in age from 35 to 81 years old. They included 125 patients with PDAC and 125 volunteers matched for age and sex. Age, gender composition, and smoking habits did not differ significantly between the groups examined, according to the demographic features of the groups. The group of PDAC had considerably higher levels of CA19-9 and CEA than controls (*p*<0.001) (Table 1). However, their levels were not associated with the clinical grading of the disease (Table 2).

**Table 1:** Demographic, laboratory, and radiological information of the examined groups.

	Control (n = 125)	PDAC (n = 125)	*P-* value
**Age (year)**	58.01 ± 0.64	59.39 ± 1.00	0.25
**Gender**			
Males	75 (60.00 %)	82 (65.60 %)	0.36
Females	50 (40.00 %)	43 (34.40 %)	
**Smoking**			
No	66 (52.80 %)	71 (56.80 %)	0.53
Yes	59 (47.20 %)	54 (43.20 %)	
**Routine Lab.**			
AST (IU/L)	26.32 ± 1.11	69.95 ± 5.64	<0.001
ALT (IU/L)	23.90 ± 1.29	76.90 ± 9.13	<0.001
Total bilirubin (mg/dL)	0.59 ± 0.02	7.95 ± 0.51	<0.001
Albumin (g/dL)	3.93 ± 0.03	3.77 ± 0.26	<0.001
Urea (mg/dL)	26.25 ± 0.84	41.23 ± 2.14	<0.001
Creatinine (mg/dL)	0.94 ± 0.02	1.20 ± 0.05	<0.001
CA19-9 (U/mL)	18.09 ± 1.06	624.85 ± 40.47	<0.001
CEA (ng/mL)	2.78 ± 0.18	221.89 ± 13.61	<0.001
**Radiological grading**			
Grade 1: Pancreatic mass with No lymphadenopathy		15 (12.00 %)	
Grade 2: Mass with enlarged regional lymph nodes, no vascular invasion		43 (34.40 %)	
Grade 3: Mass with enlarged lymph nodes and vascular, duodenal invasion, or CBD invasion		67 (53.60 %)	

Quantitative data are expressed as mean ± standard error (SE).

Qualitative data are expressed as frequencies (percentages).

Pancreatic duct adenocarcinoma (PDAC); Aspartate aminotransferase (AST); Alanine aminotransferase (ALT); Carbohydrate antigen 19-9 (CA19-9); Carcinoembryonic antigen (CEA).

**Table 2:** Levels of biomarkers and distribution of CTLA-4 genotypes across the clinical grades of PDAC.

	Clinical grading of PDAC			*P-*value
	Grade 1	Grade 2	Grade 3	
**CA19**–**9 (U/mL)**	565.78 ± 90.29	524.00 ± 53.56	702.79 ± 63.08	0.112
**CEA (ng/mL)**	207.13 ± 32.13	203.87 ± 21.68	236.76 ± 20.03	0.504
**rs231726 genotypes**				
CC	7 (46.60 %)	16 (37.20 %)	13 (19.40 %)	0.011
CT	4 (26.70 %)	17 (39.50 %)	18 (26.90 %)	
TT	4 (26.70 %)	10 (23.30 %)	36 (53.70 %)	
**rs11571317 genotypes**				
CC	12 (80.00 %)	31 (72.10 %)	52 (77.60 %)	0.66
CT	1 (6.70 %)	9 (20.90 %)	11 (16.40 %)	
TT	2 (13.30 %)	3 (7.00 %)	4 (6.00 %)	

Quantitative data are expressed as mean ± standard error (SE).

Qualitative data are expressed as frequencies (percentages).

Carbohydrate antigen 19-9 (CA19-9); Carcinoembryonic antigen (CEA).

### 3.2 Association of rs231726 with PDAC risk and clinical parameters

The three investigated CTLA-4 SNPs adhered to the Hardy-Weinberg equilibrium (*p*>0.05), indicating that our study subjects constituted a representative population.

The rs231726 CC genotype was significantly lower in PDAC patients (28.80 %) than in the healthy subjects (60.00 %). Nevertheless, the TT genotype was markedly increased in PDAC patients (40.00 %) when compared to controls (9.60 %) (*p*<0.001). Individuals with the TT genotype demonstrated a 6.28-fold elevated risk of PDAC relative to those with CC + CT genotypes, while carriers of the CC genotype had substantially less susceptibility to develop PDAC (*p*<0.001). The frequency of the C-allele was markedly diminished in PDAC patients (44.40 %) compared to the control group (75.20 %), whereas the T-allele was more common in PDAC cases (55.60 %) than in controls (24.80 %) and was associated with a 3.80-fold greater risk of PDAC (*p*<0.001) (Table 3).

**Table 3:** Genotype and allele frequencies of the rs231726 (C > T) polymorphism in the examined groups and their association with PDAC Risk.

	Control (n = 125)	PDAC (n = 125)	*P-*value	Odds ratio	Confidence interval
**Genotype**					
CC	75 (60.00 %)	36 (28.80 %)	<0.001		
CT	38 (30.40 %)	39 (31.20 %)			
TT	12 (9.60 %)	50 (40.00 %)			
**Genotype**					
CC	75 (60.00 %)	36 (28.80 %)^a^	<0.001	0.27	0.16–0.46
CT + TT	50 (40.00 %)	89 (71.20 %)^a^			
**Genotype**					
CT	38 (30.40 %)	39 (31.20 %)	0.89	1.038	0.61 - 1.78
CC + TT	87 (69.60 %)	86 (68.80 %)			
**Genotype**					
TT	12 (9.60 %)	50 (40.00 %)∗	<0.001	6.28	3.14–12.57
CC + CT	113 (90.40 %)	75 (60.00 %)^a^			
**Allele**					
C	188 (75.20 %)	111 (44.40 %)^a^	<0.001	0.26	0.18 - 0.39
T	62 (24.80 %)	139 (55.60 %)∗			

The data are listed as frequencies (percentages).

∗ Significant increase compared to the control group (*p<*0.001).

a Significant decrease compared to the control group (*p<*0.001).

**Fig. 1:**
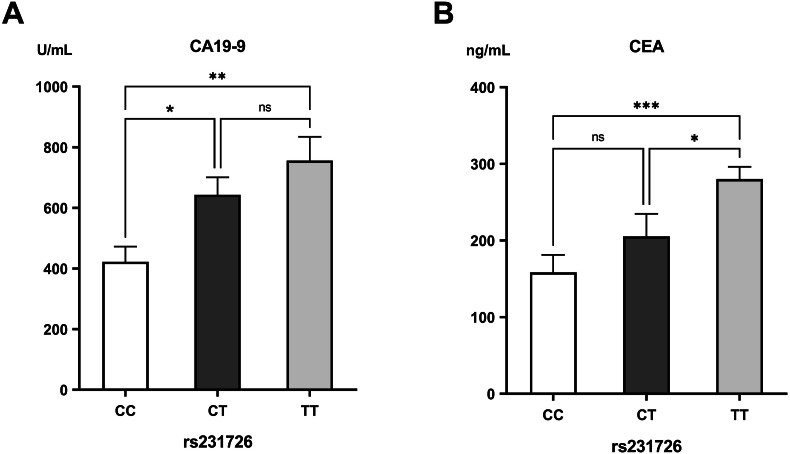
Levels of (A) carbohydrate antigen 19-9 (CA19-9) and (B) carcinoembryonic antigen (CEA) in the different genotypes of rs231726. ∗Significant difference with *p*<0.05; ∗∗significant difference with *p*<0.01; ∗∗∗significant difference with *p*<0.001.

The distribution of the rs231726 TT genotype was positively associated with the clinical grading and severity of manifestations of PDAC, as the largest proportion of patients with grade 3 had the TT genotype (53.70 %). This was contrasted with smaller percentages in clinical grades 1 and 2. Also, PDAC patients with the TT genotype had the highest levels of CA19-9 and CEA. On the other side, the CC genotype was most prevalent in patients with PDAC grade 1 (46.60 %), diminishing as the clinical grade increases, and was associated with significantly lower levels of CA19-9 and CEA (Table 2 and Fig. 1).

### 3.3 Association of rs11571317 with PDAC risk and clinical parameters

**Fig. 2:**
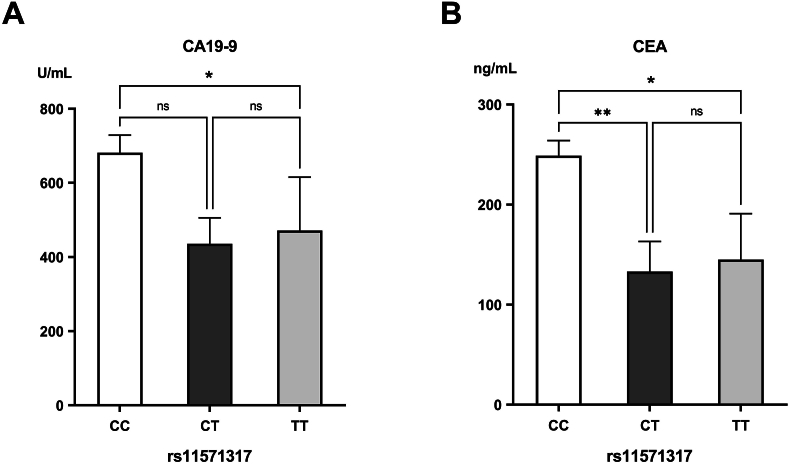
Levels of (A) carbohydrate antigen 19-9 (CA19-9) and (B) carcinoembryonic antigen (CEA) in the different genotypes of rs231726. ∗Significant difference with *p*<0.05; ∗∗significant difference with *p*<0.01; ∗∗∗significant difference with *p*<0.001.

The rs11571317 CC genotype was notably higher in the PDAC group (76.00 %) than in the controls (39.20 %). Conversely, the CT and TT genotypes were significantly reduced in PDAC cases (16.80 % and 7.20 %, respectively) compared to controls (43.20 % and 17.60 %, respectively) (*p*<0.001). In allele analysis, the C-allele was significantly more frequent in PDAC patients (84.40 %) in comparison to the control group (60.80 %) (*p*<0.001), whereas the T-allele was less represented (15.60 % in PDAC vs. 39.20 % in controls) (*p*<0.001). Individuals possessing the CC genotype and C-allele had a 4.91 and 3.49-fold increased risk of developing PDAC, respectively (Table 4). The genotypes of rs11571317 didn't reveal an association with the clinical grading of PDAC, as the CC genotype exhibited the greatest prevalence across all grades (Table 2). However, individuals carrying the CC genotype had significantly elevated levels of CA19-9 compared to those with the CT genotype (*p*=0.024) and higher levels of CEA than patients having the CT or TT genotypes (*p*<0.001 and *p*=0.043, respectively) (Fig. 2).

**Table 4:** Genotype and allele frequencies of the rs11571317 (C > T) polymorphism in the examined groups and their association with PDAC Risk.

	Control (n = 125)	PDAC (n = 125)	*P-*value	Odds ratio	Confidence interval
**Genotype**					
CC	49 (39.20 %)	95 (76.00 %)	<0.001		
CT	54 (43.20 %)	21 (16.80 %)			
TT	22 (17.60 %)	9 (7.20 %)			
**Genotype**					
CC	49 (39.20 %)	95 (76.00 %)∗∗	<0.001	4.91	2.85–8.47
CT + TT	76 (60.80 %)	30 (24.00 %)^a^			
**Genotype**					
CT	54 (43.20 %)	21 (16.80 %)^a^	<0.001	0.27	0.15–0.48
CC + TT	71 (56.80 %)	104 (83.20 %)∗∗			
**Genotype**					
TT	22 (17.60 %)	9 (7.20 %)^b^	0.02	0.36	0.16–0.82
CC + CT	103 (82.40 %)	116 (92.80 %)∗			
**Allele**					
C	152 (60.8 %)	211 (84.40 %)∗∗	<0.001	3.49	2.28–5.34
T	98 (39.20 %)	39 (15.60 %)^a^			

The data are listed as frequencies (percentages).

∗ Significant increase compared to the control group (*p<*0.05).

∗∗ Significant increase compared to the control group (*p<*0.001).

a Significant decrease compared to the control group (*p<*0.001).

b Significant decrease compared to the control group (*p<*0.05).

### 3.4 Analysis of rs13384548 and PDAC risk

The genotype and allele distribution for the CTLA-4 rs13384548 (G > A) polymorphism did not show significant differences between PDAC patients and controls. The frequency of the GG genotype was slightly elevated in the PDAC group (40.80 %) when compared to controls (31.20 %), whilst the GA (PDAC: 32.00 %, controls: 44.00 %) and AA (PDAC: 27.20 %, controls: 24.80 %) genotypes frequencies were comparable across both groups. Individuals with the GG genotype showed no significant association with PDAC risk. Similarly, the GA and AA genotypes also lacked significant associations. Allele frequency analysis further supported these findings, as the G-allele (PDAC: 56.80 %, controls: 53.20 %) and A-allele (PDAC: 43.20 %, controls: 46.80 %) distributions were similar, with no significant differences observed (Table 5).

**Table 5:** Genotype and allele frequencies of the rs13384548 (G > A) polymorphism in the examined groups and their association with PDAC Risk.

	Control (n = 125)	PDAC (n = 125)	*P-*value	Odds ratio	Confidence interval
**Genotype**					
GG	39 (31.20 %)	51 (40.80 %)	0.13		
GA	55 (44.00 %)	40 (32.00 %)			
AA	31 (24.80 %)	34 (27.20 %)			
**Genotype**					
GG	39 (31.20 %)	51 (40.80 %)	0.15	1.52	0.90 - 2.56
GA + AA	86 (68.80 %)	74 (59.20 %)			
**Genotype**					
GA	55 (44.00 %)	40 (32.00 %)	0.07	0.60	0**.**36 - 1.00
GG + AA	70 (56.00 %)	85 (68.00 %)			
**Genotype**					
AA	31 (24.80 %)	34 (27.20 %)	0.77	1.13	0.64–1.99
GG + GA	94 (75.20 %)	91 (72.80 %)			
**Allele**					
G	133 (53.20 %)	142 (56.80 %)	0.47	1.16	0.81 - 1.65
A	117 (46.80 %)	108 (43.20 %)			

The data are listed as frequencies (percentages).

### 3.5 Levels of sCTLA-4 and association with CTLA-4 genotypes

**Fig. 3:**
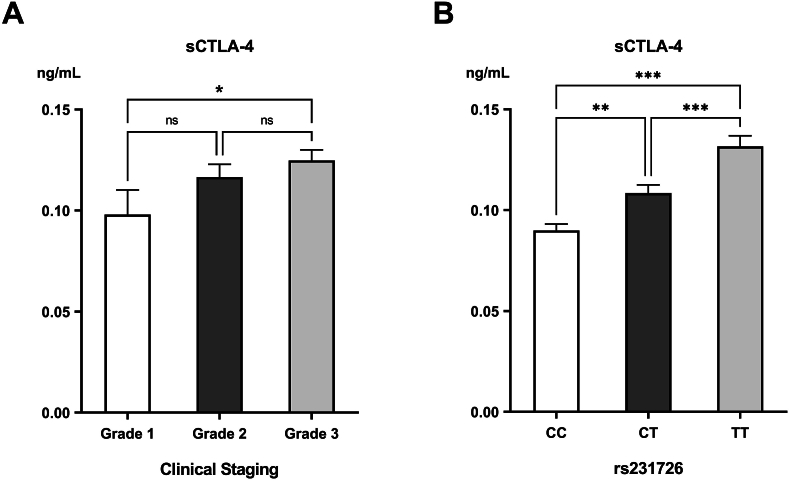
Levels of soluble CTLA-4 (sCTLA4) in the different (A) clinical grades of PDAC and (B) genotypes of rs11571317. ∗Significant difference with *p*<0.05; ∗∗significant difference with *p*<0.01.

PDAC patients had a statistically significant elevation in the serological sCTLA-4 (0.12 ± 0.004 ng/mL) relative to the control group (0.093 ± 0.003 ng/mL) (*p*<0.001). This elevation was greatest in those with grade 3 disease (Fig. 3a). The sCTLA-4 was significantly higher in those having the rs231726 TT genotype compared to those having the CC and CT genotypes (*p*<0.001) (Fig. 3b). On the other hand, the sCTLA-4 levels had no association with the rs11571317 genetic variants.

### 3.6 Correlations of sCTLA-4 levels with PC biomarkers

**Fig. 4:**
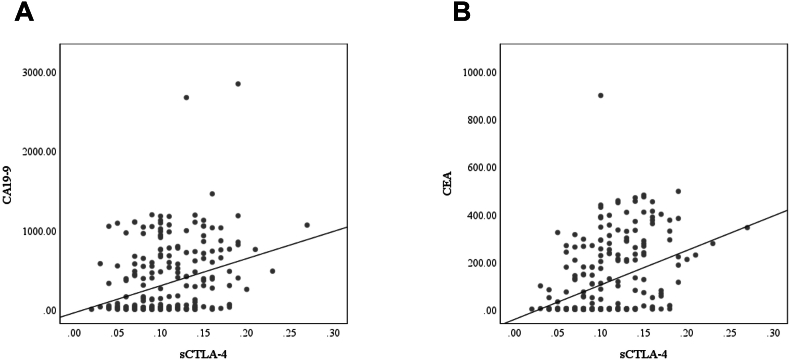
Scatterplot diagrams showing the correlations between the soluble CTLA-4 (sCTLA4) and (A) carbohydrate antigen 19-9 (CA19-9) and (B) carcinoembryonic antigen (CEA).

Spearman's correlation analysis revealed significant relationships between sCTLA-4 and CA19.9 and CEA levels in the research subjects. sCTLA-4 levels showed a weak but substantial positive correlation with CA19-9 (r = 0.267, *p*<0.001) and CEA (r = 0.371, *p*<0.001) (Fig. 4).

## 4 Discussion

This study aimed to investigate the association between the CTLA-4 genetic variants rs231726, rs11571317, and rs13384548 and the serological levels of soluble CTLA-4 (sCTLA-4) and the risk of developing PDAC in the Egyptian population. It was revealed that the rs231726 TT genotype and T-allele were associated with a 6.28- and 3.80-fold greater susceptibility to develop PDAC, respectively, whereas the CC genotype conferred protection. Also, the TT genotype was linked to advanced clinical grading, tumor aggressiveness, and elevated biomarker levels. In addition, carriers of the rs11571317 CC genotype and C-allele exhibited an increased risk of PDAC, with odds ratios of 4.91 and 3.49, respectively; however, no significant association was observed with clinical grading. The rs13384548 polymorphism had no significant correlation with the PDAC risk. Patients with PDAC had increased sCTLA-4 levels, especially those with grade 3 illness and those with the rs231726 TT genotype, whereas sCTLA-4 levels showed no correlation with the rs11571317 polymorphism. On the other side, the levels of the currently utilized biomarkers for PC diagnosis, CA19-9 and CEA, were not associated with the clinical grading and severity of the disease.

Pancreatic cancer (PC) is among the most fatal types of cancer. Unfortunately, it rarely presents early, and symptoms that necessitate diagnostic evaluations typically arise when the cancer is already advanced and has metastasized to other organs. The screening of PC can facilitate the prompt identification of small tumors and noninvasive precursor lesions in asymptomatic individuals, resulting in improved patient outcomes [21]. However, screening options for those at risk of developing PC are limited and primarily rely on CT scanning, MRI, and EUS biopsy, which are expensive and invasive for the patients. A non-invasive PC screening method involves the identification of biomarkers in blood samples. The FDA-approved biomarker CA19-9 is currently the only extensively utilized biomarker for PC diagnosis. Nevertheless, this biomarker lacks the sensitivity and specificity that are characteristic of a gold-standard serological marker. Additional biomarkers not yet approved by the FDA but under evaluation include CA-125 and CEA [4].

CTLA-4 is an inhibitory cell surface receptor that belongs to the immunoglobulin-like receptor superfamily. It is expressed by the conventional T cells upon activation and constitutively by the regulatory T cells. The human CTLA-4 exists in two main isoforms: the full-length CTLA-4 (flCTLA-4) and soluble CTLA-4 (sCTLA-4), which is devoid of the transmembrane domain [22]. CTLA-4 is regarded as the “leader” of the immune checkpoint inhibitors, as it inhibits autoreactive T cells at the initial phase of naïve T-cell activation, which typically occurs in lymph nodes [23]. It binds to the same ligands as the structurally similar CD28; CD80 (B7.1) and CD86 (B7.2), but with stronger affinity and avidity, dampening the T-cell response by blocking the co-stimulatory signals from CD28 [24]. Another mechanism by which the Tregs are thought to control the effector T cells is by down-regulating the expression of B7 ligands on the APCs, resulting in reduced CD28 costimulation [25,26].

From an oncological perspective, one key feature of CTLA-4 is its role in facilitating the immune evasion by cancer cells, as these cells utilize mechanisms that result in the overexpression of CTLA-4, thereby suppressing the immune response against them [27]. The discovery achieved by Prof. Allison's group that the CTLA-4 blockade enhances the anti-tumor immunity in a murine model, offers a novel perspective on cancer therapeutic approaches [28].

The immunological reactivity against cancer is influenced by many factors, including the patients’ immune status, which can be altered by the SNPs of immune-related genes [29]. These SNPs may reside in the regulatory regions, either impairing or introducing new binding sites for transcription factors or miRNAs, thus affecting the expression of the encoded molecules [30]. Consequently, genetic polymorphisms may disrupt the functionality of molecules critical for the efficacy of the anti-tumor response, such as CTLA-4 [27].

The rs231726 C > T is a transition substitution polymorphism in exon 1 of the CTLA-4 gene. It was identified as being linked to systemic lupus erythematosus [31] but not alopecia areata in the Jordanian population [32]. In this study, carriers of the rs231726 TT genotype and T-allele were six- and four-times more susceptible to develop PDAC, respectively, while those having the CC genotype were protected against cancer development. Also, the TT genotype was associated with advanced clinical staging and elevated levels of CA19-9, CEA, and sCTLA-4.

The promoter and 3′ UTR genetic variations have been shown to affect the expression levels of alternatively spliced flCTLA-4 and sCTLA-4 isoforms. One of these genetic variants is the rs11571317 C > T polymorphism, located in the 5’ UTR region. Previous studies on breast cancer patients found that the CC genotype was correlated with a three-fold greater risk of cancer development in the Chinese Han [33] and Moroccan populations [34]. It has been revealed that the rs11571317 polymorphism is situated in a potential binding site for specificity protein 1 (SP1) transcription factor, and that the presence of thymine at this position disrupts the SP1 binding motif. This data indicates that the T-allele is associated with reduced expression of the CTLA-4 molecule, resulting in enhanced T-cell activity and a more robust anti-tumor immune response. In turn, the C-allele, which causes higher expression of the CTLA-4 molecule, confers an increased risk of cancer development [33]. Similarly, the present study detected a five- and three-fold elevation in the risk of developing PDAC in an Egyptian population having the CC genotype and C-allele, respectively. In contrast, no association was detected between the rs11571317 and susceptibility to colorectal cancer in Saudi patients [35].

Another investigated polymorphism is rs13384548, a rare genetic variant located within the 3′-UTR of the CTLA-4 mRNA [36]. It interferes with the miRNA-302a binding site, reducing the ability to control CTLA-4 mRNA transcription. It has been demonstrated to contribute to T1D development by altering the miRNA-mediated post-transcriptional gene regulation [37]. Dissimilarly, the current study didn't detect an association between rs13384548 and the risk of PDAC. This could be attributed to the fact that the specific immune pathways modulated by this polymorphism (miRNA-mRNA interaction) are not a primary mechanism of immune suppression in the PDAC microenvironment. This result indicates that not all functionally relevant immune-related variants contribute equally to every immune-mediated disease, highlighting the disease-specific nature of genetic risk.

Accumulating evidence indicates that the tumor cells express elevated levels of sCTLA-4, and the presence of sCTLA-4 in the tumor microenvironment suppresses effector T-cell activation, presenting a novel mechanism for tumor evasion [38,39]. In this study, PDAC patients exhibited a statistically significant elevation in the serological sCTLA4 levels. This rise was highest in individuals with grade 3 illness. Likewise, sCTLA-4 has been reported to be a pivotal factor in the pathogenesis and progression of glioma, potentially serving as a significant predictive biomarker for diagnosis and evaluation of disease progression and prognosis [40]. In addition, increased expression of CTLA-4 has been established in colorectal cancer tissues compared to normal tissues [35]. CTLA-4 was overexpressed in breast tumors and invasive ductal carcinomas, with no such overexpression observed in benign breast tissues. Moreover, high expression of CTLA-4 has been an indicator of an unfavorable prognosis [41].

This study has certain limitations that warrant consideration. First, it is a single-center study, which may limit the generalizability of our findings to other ethnic groups; thus, the results require validation in multi-center and multi-ethnic cohorts. Second, the case-control design identifies associations but is incapable of determining the prognostic value of these biomarkers over time. Future longitudinal studies are needed to assess the efficacy of sCTLA-4 and CTLA-4 genotypes in predicting disease progression or treatment response. Finally, as a clinical association research, it lacks functional validation. The mechanistic links between the identified genetic variants, particularly rs231726, and the observed elevated sCTLA-4 levels remain to be elucidated through experimental models to figure out how these SNPs influence immune response in the PDAC microenvironment.

The current study reveals a compelling link between the CTLA-4 SNPs and the serological levels of sCTLA-4 with PDAC susceptibility and progression in the Egyptian population. This is, to our knowledge, the first report on these polymorphisms in patients with PDAC. Our findings underscore the importance of the rs231726 TT genotype and T-allele as effective predictors of increased PDAC risk and disease severity, while identifying the rs11571317 CC genotype and C-allele as contributors to PDAC vulnerability. Elevated sCTLA-4 levels, particularly in high-grade tumors, emphasize its potential as a prognostic biomarker. The distinct association of these biomarkers with PDAC development supports their value in non-invasive diagnostic approaches, personalized risk evaluation, and tailored therapeutic regimens. Incorporating these novel insights into the existing clinical frameworks can enable early detection of the disease, thereby improving the outcomes for individuals predisposed to this devastating malignancy. The findings of this study lay the groundwork for future studies focused on advancing precision oncology.

## Funding sources

This research did not receive any specific grant from funding agencies in the public, commercial, or not-for-profit sectors.

## Declaration of competing interest

The authors declare that they have no known competing financial interests or personal relationships that could have appeared to influence the work reported in this paper.

## Data Availability

All data generated or analyzed during this study are included in this published article.
